# *Rickettsia* sp. in *Ixodes granulatus* Ticks*,* Japan

**DOI:** 10.3201/eid1412.080894

**Published:** 2008-12

**Authors:** Hiromi Fujita, Teruki Kadosaka, Yoshiki Nitta, Shuji Ando, Ai Takano, Haruo Watanabe, Hiroki Kawabata

**Affiliations:** Ohara General Hospital, Fukushima, Japan (H. Fujita); Aichi Medical University School of Medicine, Aichi, Japan (T. Kadosaka); Okinawa Prefectural Institute of Animal Health, Okinawa, Japan (Y. Nitta); National Institute of infectious Diseases, Tokyo (S. Ando, A. Takano, H. Watanabe, H. Kawabata); Gifu University, Gifu, Japan (A. Takano)

**Keywords:** Spotted fever group Rickettsia, Ixodes granulatus, Rickettsia IG-1, letter

**To the Editor:** The genus *Rickettsia* consists of obligate intracellular bacteria that cause spotted fever and typhus fever; these bacteria are usually transmitted by an arthropod vector. We report isolation of a *Rickettsia honei–*like organism from the *Ixodes granulatus* tick; this organism may be a causative agent of rickettsiosis in Japan. Serotyping and DNA-sequencing analysis distinguished this *I. granulatus* isolate from previously reported *Rickettsia* spp.

During 2004–2005, an investigation of rickettsiosis was conducted in Okinawa Prefecture in the southernmost part of Japan, an area known to be inhabited by *I. granulatus*, a parasitic tick commonly found on small mammals. A total of 43 *I. granulatus* ticks (3 larvae, 27 nymphs, 8 adult females, and 5 adult males) were collected from small mammals (*Rattus rattus*, *R. norvegicus*, *Suncus murinus*, *Mus calori*, and *Crocidura watasei*) for the present study. For the isolation of *Rickettsia* spp., the cell line L929 was used as previously described (*1*). A total of 13 isolates, designated as strains GRA-1 to GRA-13, were obtained from 11 ticks (1 fed larva, 5 fed nymph, 1 fed adult female, 1 fed adult male, 1 unfed nymph molted from engorged larva, 2 unfed adult females molted from engorged nymphs) and from 1 pool of eggs and 1 larva derived from the engorged female tick.

Serotyping was performed by using a microimmunoperoxidase approach according to the method described by Philip et al. (*2*); we used anti-*Rickettsia* mouse serum and several spotted fever group *Rickettsia* antigens: 2 of the present isolates (GRA-1 and GRA-2) and 6 known members of the Asian *Rickettsia* spp. (*R. honei*, *R. japonica*, *R. asiatica*, *R. tamurae*, *R. sibirica,* and *R. conorii*). Differences among antigen reaction titers were calculated, and the results are given as the specificity difference (SPD) value. The SPD value between the present isolates and *R. honei* was 0 or 1, whereas the SPD values were >3 for the other spotted fever group *Rickettsia* spp. (Table). According to the criteria for serotyping (*2*), we assumed the isolates to be of the same serotype when the SPD value was <2. In addition to serotyping, a sequencing analysis was performed to genetically characterize the isolates. The archive of DNA sequences has been mostly established for the outer membrane protein A gene (*ompA*), citrate synthesis gene (*gltA*), and 17-kDa antigen gene. Thus, we determined these DNA sequences in the isolates and compared the results with those of representative *Rickettsia* spp. The *ompA* sequencing analysis showed a DNA sequence of 491 bp in the 6 isolates from *I. granulatus* (GenBank accession nos. AB444090–AB44095), which yielded the following similarity values: *R. slovaca* (98.0%), *R. honei* and Thai tick typhus *Rickettsia* (97.8%), and *R. honei* subsp. *marmionii* (97.6%). Sequencing of the 1,250-bp fragment of *gltA* of the strain GRA-1 (accession no. AB444098) showed >99% DNA similarity with that of *R. sibirica* (99.3%), *R. slovaca* (99.2%), *R. conorii* (99.2%), *R. honei* (99.1%), and certain types of *Rickettsia* spp. Moreover, 17-kDa antigen gene sequencing analysis of a 392-bp fragment of the strain GRA-1 (accession no. AB444097) showed the highest levels of sequencing similarity value with *R. honei* and Thai tick typhus *Rickettsia* (99.5%) compared with those of the sequences of other deposited *Rickettsia* spp. Comprehensive analyses led us to presume that the isolate GRA-1 from *I. granulatus* was a genetic variant of *R. honei*, although further studies are necessary to better define the taxonomic position of our isolates.

**Table:** Serotype results for *Rickettsia* sp. strains GRA-1 and GRA-2 from *Ixodes granulatus* tick, Okinawa Prefecture, Japan

Mouse antiserum to	Results*							
	GRA-1	GRA-2	TT-118	Aoki	IO-1	AT-1	246	Moroccan
Strains from this study								
*Rickettsia* sp., GRA-1	**320†**	**320** (0)‡	160 (1)	80 (3)	40 (6)	40 (6)	80 (4)	80 (3)
*Rickettsia* sp., GRA-2	**320** (0)	**320**	**320** (0)	40 (4)	20 (7)	40 (7)	40 (5)	40 (5)
Reference strains								
*R. honei*, TT-118	**5,120** (1)	**5,120** (0)	**5,120**	80 (7)	320 (7)	80 (9)	160 (7)	80 (7)
*R. japonica*, Aoki	2,560 (3)	2,560 (4)	1,280 (7)	**5,120**	320 (9)	320 (10)	320 (9)	32 0 (8)
*R. asiatica*, IO-1	640 (6)	640 (7)	80 (7)	160 (9)	**5,120**	80 (12)	80 (11)	160 (11)
*R. tamurae*, AT-1	640 (6)	320 (7)	80 (9)	80 (10)	80 (12)	**5,120**	160 (11)	40 (13)
*R. sibirica*, 246	1,280 (4)	1,280 (5)	320 (7)	160 (9)	160 (11)	80 (11)	**5,120**	320 (7)
*R. conorii*, Moroccan	640 (3)	640 (5)	80 (7)	80 (8)	20 (11)	20 (13)	160 (7)	**1,280**

*Highest serum dilutions against each *Rickettsia* antigen (specificity difference between each pair of strains), determined by microimmunoperoxidase method. **Boldface** indicates equivocal titer to homologous antigen. †Highest serum dilution showing a positive reaction with antigen. ‡Specificity difference between each pair of strains.

The vector for *R. honei* was presumed to be ixodid ticks: *I. granulatus* in Thailand; *Amblyomma cajennense* in Texas, USA; and *Aponomma hydrosauri* in Australia (*3**–**5*). Lane et al. reported that a *Rickettsia* organism from a *Haemaphysalis* tick was closely related to *R. honei* in Australia (*6*). In the present study, we observed that the *Rickettsia* organism was maintained in the tick after molting. Moreover, *Rickettsia* organisms were also isolated from egg and unfed larva. These preliminary findings may suggest that *I. granulatus* is a possible vector for the *R. honei–*like bacterium in Japan.

Recently, a *Rickettsia* sp. was found in *I. granulatus* ticks; its proposed designation was unclassified *Rickettsia* IG-1, according to DNA sequencing from specimens obtained in Taiwan (*7*). With respect to the DNA sequences of *gltA* and *ompA*, our isolates from *I. granulatus* were identical to the *Rickettsia* IG-1.

*R.* *honei*, a member of the spotted fever group *Rickettsia,* has been reported as the etiologic agent of Flinders Island spotted fever in Australia (*8*) and also of Thai tick typhus (*3*). *R. honei* is a public health threat for rickettsiosis in these countries. Although the human health implications of the *Rickettsia* sp. found in this study are not yet known, knowledge from this study will be useful in epidemiologic investigation for rickettsiosis in Japan.
